# Benefits of targeted deployment of physician-led interprofessional pre-hospital teams on the care of critically ill and injured patients: the ‘science’ explained

**DOI:** 10.1186/s13049-025-01398-z

**Published:** 2025-05-01

**Authors:** Michael D. Christian, Matthew D. Lavery, Arshbir Aulakh

**Affiliations:** 1 Faculty of Medicine, Department of Critical Care Medicine, University of British Columbia, Vancouver, Canada; 2 Southern Medical Program, Faculty of Medicine, University of British Columbia, Kelowna, Canada

We appreciate the opportunity to respond to Davis et al.’s critique [1] of our recent systematic review and meta-analysis [2]. Their commentary misrepresents both our methodology as lacking rigour and discounts the broader body of evidence our meta-analysis draws upon. Hence, their assertion that our findings may lead to misguided clinical and policy decisions is unfounded. Below, we respond point-by-point to the concerns raised including the role of intention to treat (ITT) vs as treated (AT) analyses within the Head Injury Retrieval Trial (HIRT), the reliance upon randomized controlled trials (RCTS), the role of observational studies and managing heterogeneity in meta-analyses, misinterpretations around causality, and the evolving role of inter-professional teams in prehospital critical care.

## Clarifying the findings and context of the Head Injury Retrieval Trial (HIRT)

Davis et al. cite the randomized controlled trial (RCT) by Garner et al. [3] as the data that should be primarily relied upon while discounting the value of the rigorous findings of the multiple observational studies included in the meta-analysis. However, several key facts about this study appear to have been omitted or misrepresented in their commentary:

A) Early termination diminished the power of the study: The HIRT trial was stopped early due to recruitment challenges following a change in the ground ambulance service deployment strategy, which began incorporating prehospital physician response. The ambulance service's decision to adopt physician-led teams before the study results were available likely speaks to the benefits of prehospital physicians. This context is essential to understanding why the study was underpowered and why the statistical approach and findings must not be interpreted superficially, as Davis et al. appear to have.
B) Favourable intention-to-treat (ITT) findings: While not deemed "statistically significant" due to insufficient power, the ITT analysis revealed an 11% improvement in outcomes (OR 1.11) favouring the physician-led interprofessional teams—an important signal toward benefit rather than futility. However, as discussed further below, more sophisticated clinicians and researchers are increasingly cautious not to overly rely on the p-value to indicate the presence or absence of clinical importance.
C) As-treated analysis (AT) was indicated and demonstrates effectiveness: The introduction of physicians into the ground response caused substantial contamination of the control arm with one in ten patients receiving care from physician-led interprofessional teams. The AT analysis, which accounts for this, demonstrated a 16% improvement in outcomes (p<0.01) for patients treated by physician teams versus standard care, with a number needed to treat (NNT) of 6. When significant contamination (or ‘cross-over’) occurs within the control group an AT analysis will demonstrate the effectiveness of the intervention, in this case, physician-lead interprofessional teams. In such cases, researchers should conduct ITT and AT analyses and then compare and analyze any differences in results [4, 5].

These details are important, and failure to take them into consideration results in a skewed representation of the actual evidence and undermines the assertion in the commentary [1].

## An over-reliance on randomized controlled trials

Davis et al.'s insistence on RCTs as the only valid evidence source is a dated and overly rigid approach to evidence appraisal. An over-reliance on RCTs and p-values has been widely critiqued as a simplistic approach to complex healthcare questions. [6–9] Decision-making in clinical policy must integrate a broader evidence base, including real-world data and context-sensitive outcomes. As we discussed in our article, conducting another RCT in this space is highly unlikely due to logistical, ethical, and financial challenges. The issue of equipoise—both scientific and clinical—is increasingly recognized as a limiting factor in trauma research [6]. Increasingly, the scientific community acknowledges that well-conducted observational studies and quasi-experimental designs such as the implementation of inter-professional teams to the air ambulance systems in Wales [10] and Scotland [11] offer valuable insights, particularly when RCTs are impractical. [7] Although not feasible for inclusion in the meta-analyses, independent third-party evaluations of the implementation of both the Welsh and Scottish systems provide further evidence of both the clinical benefit to patients of physician-led interprofessional teams as well as their cost-effectiveness and broader impacts on recruitment, retention, health system capacity building and mitigation of health inequities [12, 13].

## Understanding and managing heterogeneity in meta-analysis and adjusting for confounders

Davis et al. [1] raise concerns that heterogeneity in our analysis weakens our conclusions. We disagree. In our analysis, we employed Cochrane-recommended methods to explore and mitigate heterogeneity, including using a random-effects model and subgroup analyses [14, 15]. However, this critique reveals a misunderstanding of how heterogeneity is handled in systematic reviews. As Cochrane guidance explicitly states, “Heterogeneity is to be expected in a systematic review of studies that differ in populations, interventions, outcomes and settings” [3]. This is not a flaw but a characteristic of complex real-world data. While a higher I^2^ measure of heterogeneity is less desirable, it does not invalidate the results, nor does a low I^2^ score mean there is no important heterogeneity at play [14–17].

**Fig. 1:**
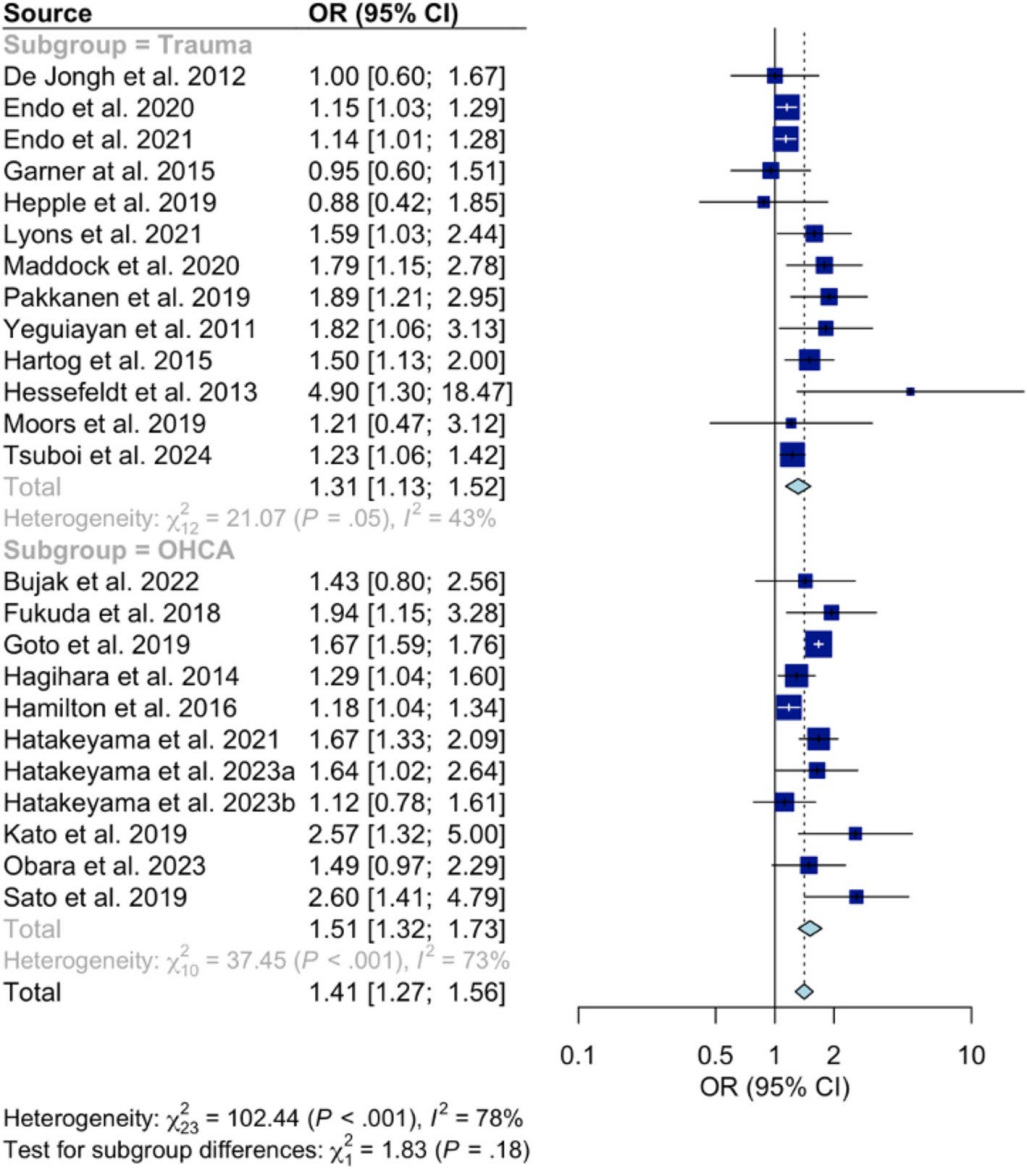
A forest plot showing the survival outcomes in patients receiving physician-based care compared to standard care (From McHenry RD. SJTREM. 2025 Mar 3;33(1):36.)

**Fig. 2:**
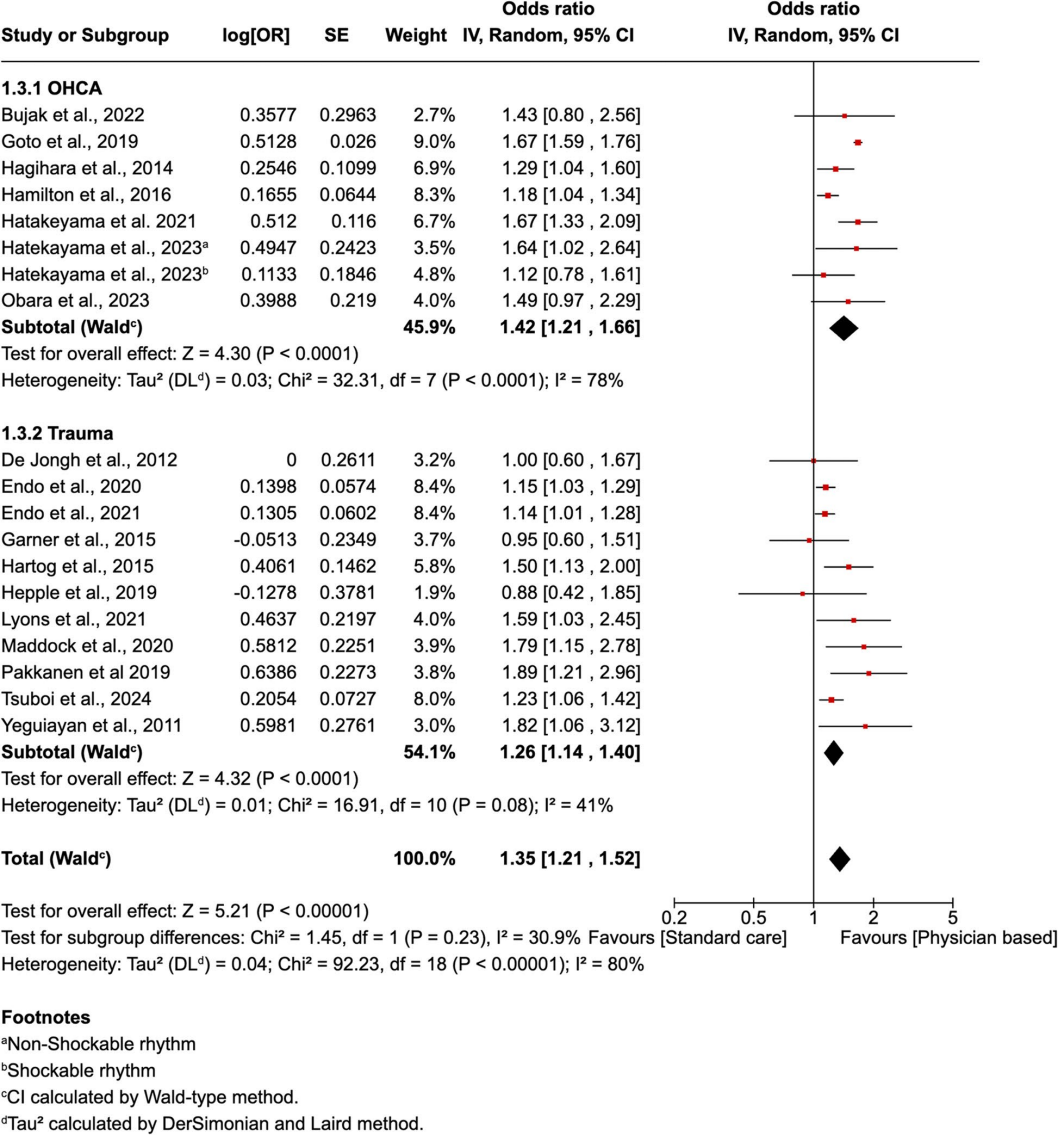
A forest plot presenting the results of a sensitivity analysis with the five most heterogeneous studies excluded

The heterogeneity observed would be more concerning if there were few studies with a single large study among them driving the results. Applying the ‘eye-ball’ test [18] using data from McHenry’s independent analysis [19] (Fig. 1) shows a general degree of homogeneity with all but three of the twenty-four studies in this analysis favouring physician-led interprofessional teams with largely similar magnitude of effect and generally overlapping confidence intervals. However, to further evaluate any potential concerns around heterogeneity, we have undertaken a post-hoc sensitivity analysis in which we excluded 20% of studies (5) with the widest 95% confidence intervals [20], and recalculated the meta-analyses results (Fig. 2). This demonstrated that the finding for survival remained both clinically and ‘statistically’ significant, although the OR decreased slightly from 1.41 to 1.35, indicating a sustained improvement in survival for patients treated by physician-led interprofessional teams of at least 35%. These results suggest that our original findings are reliable, and all studies were appropriately included as per our original inclusion criteria [2].

Further, heterogeneity within the meta-analysis does not negate the positive findings of the methodologically rigorous individual cohort and population-level studies included in the meta-analyses. The rigor of our findings is also bolstered in the comment provided by Boulton et al. [21], who found remarkably similar results in a near-simultaneously published meta-analysis in which 16 of the 17 studies included physician-led teams, thus driving the results in their analysis [22].

Finally, Davis et al. mistakenly stated that the studies included in the analysis failed to adjust for confounders consistently and that only 39% of the studies used validated physiologic scoring such as the injury severity scores (ISS), Trauma and Injury Severity Score (TRISS), or Revised Trauma Score (RTS). The ISS, TRISS and RTS are only applicable to trauma patients, not medical patients, and at least one of these was included in all but one of the thirteen studies (92%) involving trauma patients. Further, all (100%) of the observational studies included in the meta-analysis adjusted or controlled for relevant potential confounders in their original peer-reviewed, published analyses.

## Misinterpretation of causality and confounders

Davis et al.’s argument that our findings "attempt to establish causality" mischaracterizes the nature of our conclusions. The improved outcomes in systems using physician-led interprofessional teams are an unequivocal observational finding. Causality refers to explaining *why* this occurred, not *whether* it occurred.

We recognize that research on team composition and performance is complex, and in our manuscript, we articulated several plausible hypotheses behind the observed effect, including enhanced decision-making, advanced procedural capabilities, and synergistic interprofessional team effects. Thus, we did not infer or suggest causality.

We agree with Davis et al. regarding the potential impact of confounders in many prior studies in prehospital and retrieval medicine. For that reason, as we detailed in our methods, we specifically excluded studies which focused on the mode of transport rather than the model of care provided. The helicopter emergency medical services (HEMS) teams included in this study generally responded by both ground and air and transported patients to the hospital by both ground and air. In many physician-led interprofessional team-based HEMS systems, the primary aim of the aircraft is to transport the team rapidly to the patient, allowing larger coverage areas, with patients and the team frequently being conveyed to the hospital by ground rather than air. Unfortunately, specific details of the breakdown of the mode of transport to the hospital were rarely provided in the studies.

## The role of interprofessional teams and paramedics

Finally, we wish to emphasize that although our findings support the view that interprofessional teams, combining the strengths of physicians and paramedics, nurses, and other health professionals, achieve the best outcomes, this does not lessen the importance of any individual profession. There is compelling evidence across multiple healthcare domains that interprofessional collaboration improves safety, efficiency, and patient-centred outcomes [20, 23, 24]. The synergistic model, where paramedics and physicians operate in concert, holds the potential to mirror what has been seen in other aspects of healthcare systems worldwide. As stated by Kennedy et al. “*The needs of the patient and the systems outweigh professional turf, prejudice or entrenched methods, with modern approaches to quality and safety and best outcomes now being established as our key drivers. Tolerance of compromised systems, which are based on history, culture or professional posturing, is no longer acceptable*.” [25]

## Conclusion

In view of the above response to Davis et al., we suggest that our study's conclusions stand on solid grounds. We invite readers and policymakers to consider this growing body of evidence holistically and to support models of care that demonstrably improve patient outcomes.

## Data Availability

No datasets were generated or analysed during the current study.
